# Utilising Xsens Gait Analysis to Improve Functional Outcomes in Chronic Ankle Instability: A Multifaceted Physiotherapy Approach

**DOI:** 10.7759/cureus.68880

**Published:** 2024-09-07

**Authors:** Nikita Gangwani, Deepali S Patil, Gurjeet Kaur

**Affiliations:** 1 Musculoskeletal Physiotherapy, Ravi Nair Physiotherapy College, Datta Meghe Institute of Higher Education & Research, Wardha, IND

**Keywords:** balance training, chronic ankle instability, gait analysis, pain reduction, physiotherapy, rehabilitation

## Abstract

Chronic ankle instability (CAI) is a common consequence of lateral ankle sprains, resulting in persistent pain, instability, and functional limitations. This case report investigates the effectiveness of a physiotherapy intervention for a 25-year-old female patient with CAI, marked by recurrent ankle sprains and persistent symptoms despite prior conservative treatments. The patient exhibited swelling, pain, and instability, with clinical assessment revealing significant ligament laxity and a high longitudinal arch in both feet. A comprehensive physiotherapy regimen focused on core, hip, and ankle muscle strength, dynamic balance, and proprioception was implemented, incorporating ankle stretches, joint mobilization, core strengthening, hip strengthening, and dynamic balance exercises on unstable surfaces. Pre-rehabilitation outcome measures included a numeric pain rating of 7/10, a Cumberland ankle instability tool (CAIT) score of 15/30, and a foot and ankle outcome score (FAOS) of 63%. Gait analysis revealed a speed of 0.79 m/s, a cadence of 99.24 steps/min, and a distance of 14.23 meters. Post-intervention, significant improvements were observed: pain reduced to 1/10, the CAIT score increased to 28/30, and the FAOS rose to 89%. Gait parameters also improved, with speed increasing to 0.90 m/s and distance to 15 meters. This case underscores the effectiveness of a targeted physiotherapy approach in managing CAI, highlighting the importance of a multi-dimensional rehabilitation strategy to enhance functional outcomes and reduce associated symptoms of CAI.

## Introduction

Ankle sprains are common in sports and everyday life [1]. Lateral ankle sprains are a common injury, particularly in active individuals, often leading to persistent pain, limited function, and significant economic impact [2]. Chronic ankle instability (CAI) occurs in 25% of people, with a range of 7% to 53%. This rate jumps to 46% in individuals with a history of ankle sprains, varying from 9% to 76% [3]. Lateral ankle sprains are common, often resulting in chronic instability. The anterior talofibular ligament (ATFL) is the primary stabilizer against inversion and plantarflexion, while the calcaneofibular ligament (CFL) supports both ankle and subtalar joint stability. The posterior talofibular ligament (PTFL) provides additional support, especially in dorsiflexion [4].

Despite initial treatment like taping or bracing and physical therapy, up to 40% of people with lateral ankle sprains develop chronic ankle instability. This suggests that we still do not fully understand what factors predict the success of rehabilitation [5]. CAI leads to ongoing issues like pain, swelling, feeling like the ankle will give way, repeated injuries, and limited joint movement, which significantly impact a person's quality of life and financial situation [6]. Non-surgical management of lateral ankle instability primarily focuses on strengthening the ankle, core, and gluteal muscles to improve dynamic stabilization. Ankle braces or taping can provide external support [7].

While traditional treatment emphasized direct ankle rehabilitation, current research highlights the importance of a holistic approach, including core and gluteal strengthening. Biologics like platelet-rich plasma (PRP) show promise for pain relief but lack conclusive evidence. If conservative measures fail after three to six months, surgical intervention, typically involving ligament reconstruction, is considered. Anatomic reconstruction techniques generally yield better outcomes than non-anatomic approaches, although optimal surgical methods remain debated due to insufficient comparative studies [7]. Balance training is a common approach for treating ankle instability, improving both balance and body awareness. While exercises for strength, balance, and proprioception are beneficial, there is a lack of specific exercise programs for this condition [8].

This study aims to evaluate the impact of ankle-strengthening exercises on unstable surfaces to improve balance and body awareness in young adults with CAI. The goal is to strengthen ankle ligaments and muscles and improve body awareness to enhance overall balance and walking ability.

## Case presentation

Patient’s information

A 25-year-old female patient presented to the outpatient department (OPD) in July 2024, reporting swelling on the dorsum and lateral aspects of her foot, along with pain during walking and any movement of the ankle joint. Her medical history indicates that she was asymptomatic until April 2020, when she experienced her first episode of an ankle sprain. This initial injury was followed by recurrent sprains occurring at intervals of seven months, eight months, 11 months, and, most recently, within the past month in 2024. These episodes are detailed in the accompanying timeline in Table 1.

**Table 1:** Timeline of events

Date	Event	Description
April 2020	First ankle sprain	The patient experienced an inward twist of her right ankle while descending a ramp. Sought medical care, applied ice, and bandaged the area, leading to pain and swelling reduction within a week.
November 2020	Second ankle sprain	Another ankle sprain occurred while descending stairs. Treatment included icing, bandaging, ankle binders, and analgesics. Symptoms improved in 15 days, but she began to feel instability and her ankle "giving way".
July 2023	Third ankle sprain	Sustained a sprain with significant swelling and severe ligament laxity. A positive anterior drawer test confirmed this. Received physiotherapy treatment, which alleviated pain and swelling within a month.
June 2024	Latest ankle sprain	Slipped on a wet floor, leading to a sprain of the anterior talofibular ligament.
July 2024	Current symptoms	Presenting symptoms of swelling on the dorsum and lateral side of the foot, with pain during walking and ankle joint movement. Noted high longitudinal arch in both feet, possibly contributing to recurring injuries.

Clinical presentation

Before the evaluation, the patient's verbal consent was obtained. The patient appeared alert, cooperative, and oriented to time, place, and self. Her vital signs were stable, and she had a mesomorphic body type. The assessment was conducted in a long sitting position, where observation revealed swelling on the lateral aspect of the foot and dorsum. The patient exhibited high arches in both feet, with no noticeable skin colour changes or muscle wasting around the ankle and tibiofibular joint.

Upon palpation, the swelling was tender to the touch, with a grade 3 tenderness, and the patient reported a pain score of 7/10 on the numeric pain rating scale (NPRS). The joint was hypermobile and presented with a painful, soft end feel, indicating ligament laxity. A motor examination was performed, assessing joint range of motion and manual muscle strength and conducting special tests to confirm ligament laxity and the presence of an ankle sprain.

**Figure 1:**
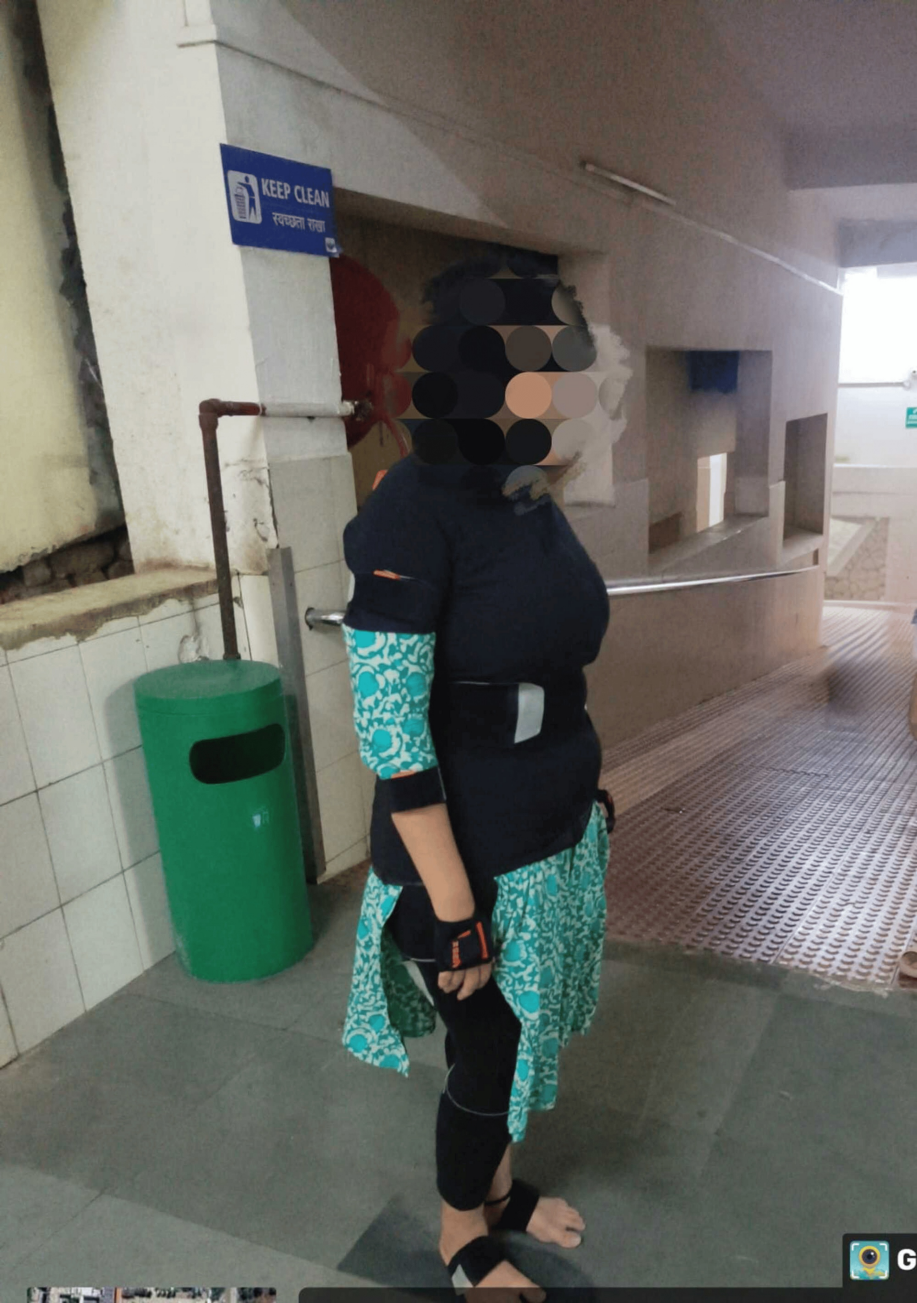
Gait analysis of the patient using Xsens sensors for gait and motion analysis

The following tables provide comparative data on the ankle and subtalar joint range of motion, as well as manual muscle testing results for the ankle joint muscles. Special tests, including the anterior drawer test, eversion stress test, and talar tilt test, were employed to aid in differential diagnosis. For gait analysis, the Xsens system was utilized. This detailed data offers clinicians valuable insights into the efficacy of the rehabilitation program, enabling necessary adjustments to optimize patient progress. The range of motion for the ankle joint is presented in Table 2. Figure 1 shows patients undergoing gait analysis while wearing Xsens gait and motion sensors.

**Table 2:** Range of motion

Joint movement	Baseline data	Post physiotherapy (four weeks)
Dorsiflexion	0-10º	0-20º
Plantar flexion	0-20º	0-35º
Inversion	0-10º	0-25º
Eversion	0-7º	0-10º

The manual muscle testing (MMT) of the muscles surrounding the hip and knee joints was evaluated using the modified medical research council (MMRC) scale and summarized in Table 3.

**Table 3:** Manual muscle testing 0: No movement, no muscle contraction observed or felt; 1: Slight muscle contraction is felt, or the tendon becomes prominent, but no visible movement; 2-: Partial range of motion is achieved; 2: Full range of motion is achieved for the muscle being tested;2+: Holds position against slight pressure and moves through partial range of motion against gravity; 3-: Gradual loss of position during testing; 3: Holds the test position with no added pressure; 3+: Maintains the test position against slight pressure; 4-: Maintains the test position against light to moderate pressure; 4: Maintains the test position against moderate pressure; 4+: Maintains the test position against moderate to strong pressure; 5: Maintains the test position against strong pressure (Normal)

Joint movements	Baseline data	Post rehabilitation (2 weeks)	Post rehabilitation (4 weeks)
Dorsiflexion	3/5	4/5	5/5
Plantar flexion	3+/5	4+/5	5/5
Inversion	3/5	4/5	5/5
Eversion	3/5	4/5	5/5

Table 4 provides detailed results and descriptions of special tests used to evaluate ankle ligament integrity, including the anterior drawer, eversion stress, and talar tilt tests. These findings are essential for diagnosing ligament injuries and informing treatment strategies.

**Table 4:** Special test ATFL: Anterior talofibular ligament; CFL: Calcaneofibular ligament

Special test	Description
Anterior drawer test	Used to assess the integrity of the ATFL. The patient sits or lies supine with the knee bent at 90° and the ankle slightly plantarflexed (10-20°). A gentle forward force is applied to the subtalar joint. To isolate the CFL, the ankle is dorsiflexed (raised upward). This test was positive.
Eversion stress test	The patient sits, lies supine, or lies on their side with the knee bent at 90° and the foot relaxed. One hand stabilizes the shinbone while the other tilts the heel outward, moving the ankle bone. Increased tilt or pain near the inner ankle compared to the opposite side may indicate a deltoid ligament issue. This test was used to rule out a sprain or tear in the deltoid ligament and was negative.
Talar tilt test	This test is to rule out CFL integrity; the patient sits or lies supine with the knee straight. The examiner steadies the lower leg while the other hand inverts the foot at the ankle. Pain, clicking, or excessive inward movement compared to the other side suggests a CFL tear. This test was found negative.

Physiotherapy intervention

The rehabilitation program begins with a structured warm-up phase that includes ankle stretches and joint mobilization exercises, followed by standing holds for ankle dorsiflexion and plantar flexion. The main exercise routine lasts 50 minutes, emphasizing core, hip, and ankle muscle strengthening through specific exercises. Dynamic balance activities are also incorporated, progressively increasing in difficulty to enhance stability. Additionally, foot core exercises and perturbation drills are included to boost proprioception and functional movement. The session concludes with a cool-down phase featuring stretches to aid in recovery. These combined interventions are designed to improve the patient's strength, balance, stability, and overall ankle function, as outlined in tables 5 and 6.

Table 5 outlines the exercise program, detailing the duration assigned to each phase, including the warm-up, core strengthening, dynamic balance, and cool-down. This breakdown provides a clear understanding of the structured progression throughout the rehabilitation regimen.

**Table 5:** Outline of exercise regimen

Intervention	Dosage
Warming up: Ankle stretches, joint bareness,s tanding ankle dorsiflexion and plantar flexion	3 sets 30 seconds hold
Exercise regimen	For 50 min
Cool down- Standing wall pushing, stretching	3 sets 30 seconds hold

**Figure 2:**
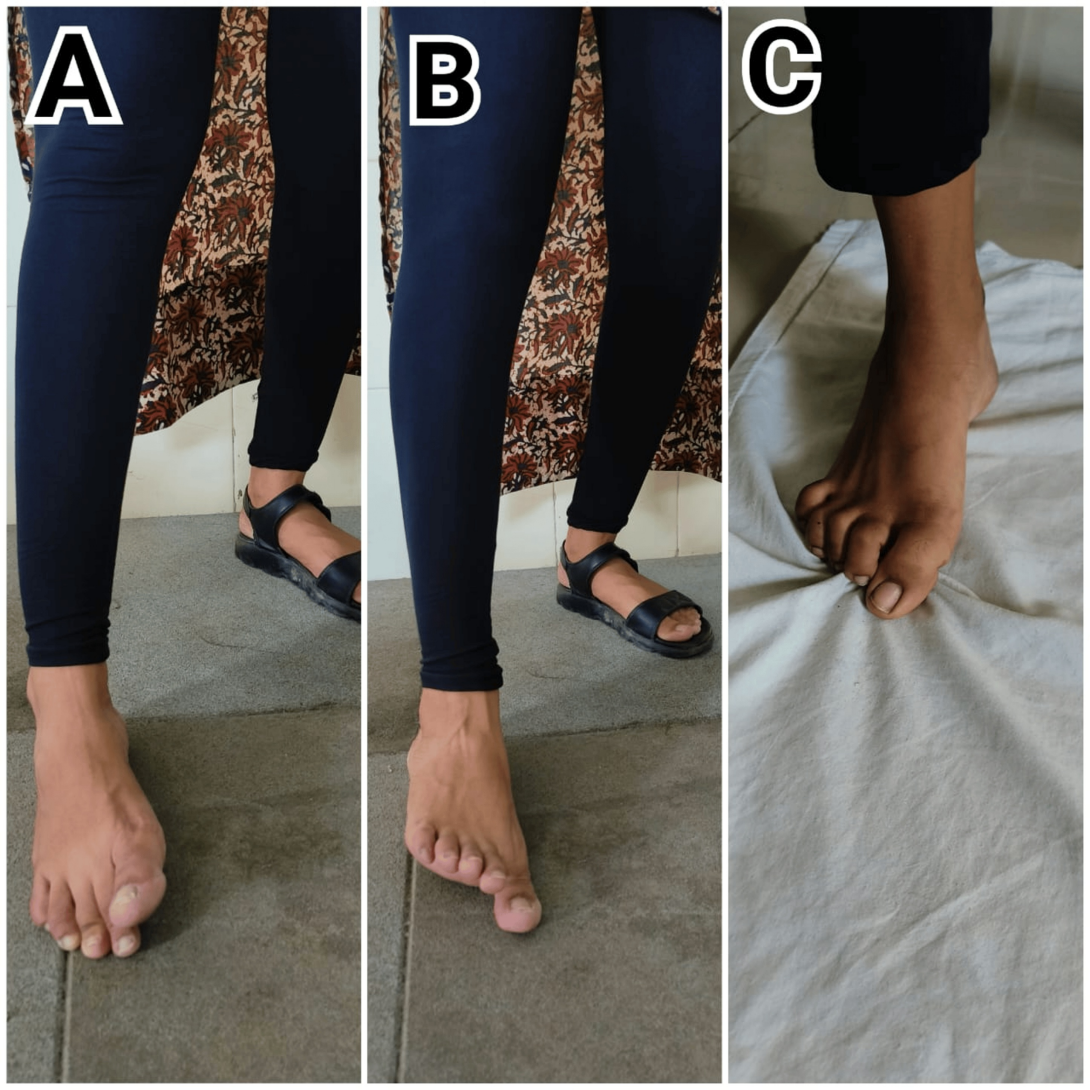
Rehabilitation protocol A: finger press and finger lift exercise, B: toe press and finger lift exercise; C: toe curls on towel

**Figure 3:**
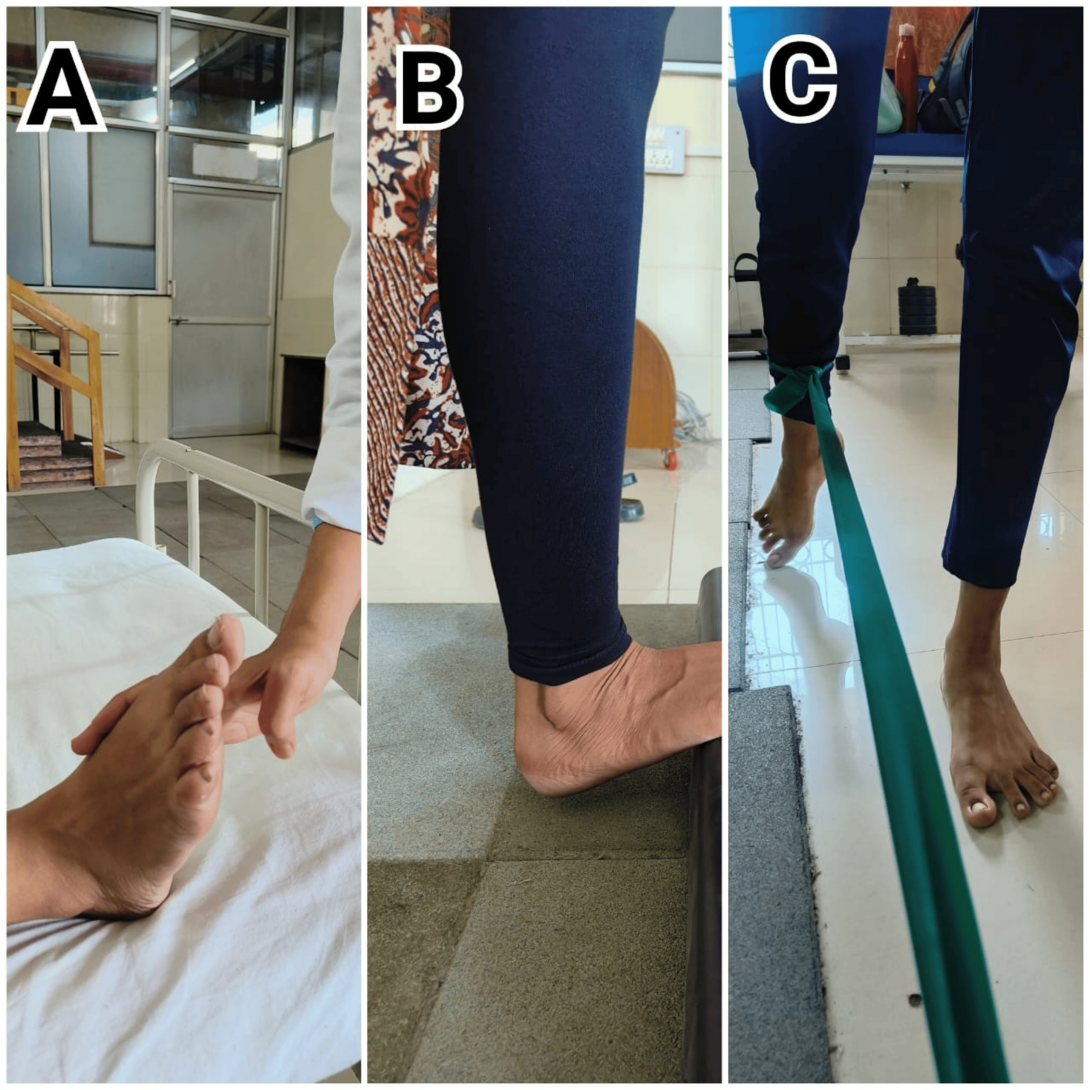
Rehabilitation protocol A: perturbations to the foot; B: plantar stretch; C: hip extension exercise with the theraband

Table 6 details the types of exercises, their dosage, and their significance in the rehabilitation program. This table highlights how specific exercises are prescribed, along with their frequency and duration, to target key areas of recovery and optimize patient outcomes. Figure 2A-2C shows rehabilitation exercise to strengthen foot intrinsic muscles, and Figure 3A-3C shows rehabilitation exercise for hip strengthening and perturbation.

**Table 6:** Rehabilitation protocol Reps: repetition; Sec: seconds

S.No	Intervention	Types	Dosage	Rationale
1.	Core strengthening	Core tucking in all fours	10 reps x 10 sec hold x 2 sets	These exercise helps to improve strength of core muscles which will eventually help in postural control
		Bird dog	10 reps x 2 sets	
2.	Hip strengthening	Hip flexion	10 reps x 2 sets	These exercises help to improve strength of hip muscles which helps in improving balance.
		Hip extension	(Progression- different color/resistance theraband)	
		Hip abduction		
3	Ankle muscle strengthening	Dorsiflexion, plantar flexion, inversion, eversion.	10 reps x 2 sets	These exercise helps to improve strength of ankle muscles which helps in improving balance and stability
		Heel Raise	(progression- different color/resistance theraband)	
		Forefoot raise		
4	Dynamic balance	Single limb stance	Single limb stance with eyes close-60sec, the progress to hard surface to foam pads for 30-60-90 sec.	These exercise helps to improve balance and stability on uneven surface
5	Foot core and perturbation	Towel curls	10 reps x 2 sets	These exercises help to improve strength of foot intrinsic muscles and improve proprioception and joint sense position.
		Foot doming	10 reps x 2 sets	
		Toe spreads	10 reps x 2 sets	
		Toe press and finger lifts	10 reps x 2 sets	
		Perturbations/rhythmic stabilization	In different position of ankle 6-10 perturbations.	

Outcome measure

The results of the rehabilitation program demonstrated significant improvements across various outcome measures. The patient's numeric pain rating decreased from 7/10 pre-rehabilitation to 1/10 post-rehabilitation, indicating a marked reduction in pain. The Cumberland ankle instability tool (CAIT) score improved substantially from 15/30 (50%) to 28/30 (93%), reflecting enhanced stability and confidence in the ankle. Similarly, the foot and ankle outcome score (FAOS) increased from 63% to 89%, highlighting an overall improvement in functional status and quality of life. In terms of gait parameters, the patient's walking speed improved to 0.90 m/s from the pre values 0.79 m/s, with a cadence of 99.48 steps per minute, taking 15 seconds to complete the task, suggesting enhanced mobility and efficiency. These results underscore the effectiveness of the rehabilitation interventions in addressing the patient's chronic ankle instability and associated symptoms. The outcome measures and gait parameter values are shown in Tables 7-8.

**Table 7:** Outcome measures NPRS: Numerical pain rating scale; CAIT: Cumberland ankle instability tool; FAOS: Foot and ankle outcome score

Outcome measures	Pre-rehabilitation	Post-rehabilitation
NPRS	7/10	1/10
CAIT	15/30 (50%)	28/30 (93%)
FAOS	63%	89%

Table 8 describes about the gait analysis, which was taken before and after the rehabilitation.

**Table 8:** Gait analysis taken with the help of Xsens

Gait Parameters	Normal values	Pre-rehabilitation values	Post-rehabilitation values
Speed (m/s)	1.2 to 1.4	0.79	0.90
Cadence (steps/min)	100 to 120	99.24	99.48
Steps	10 to 15	29	35
Duration (s)	7 to 9	17.53	15

## Discussion

This case report compellingly illustrates the transformative impact of a comprehensive physiotherapy program on a young female patient suffering from CAI. The significant improvements observed throughout the rehabilitation process highlight the efficacy of the therapeutic interventions and underscore the importance of addressing the multifaceted nature of CAI. At the outset, the patient presented with debilitating symptoms, including recurrent sprains, pain, swelling, and a pronounced sense of instability [9]. Detailed assessments revealed underlying issues such as ligament laxity, high longitudinal arches, and limited range of motion, all contributing factors to her condition [10]. Recognizing these complexities, the rehabilitation strategy employed a well-rounded approach that targeted the root causes of her ankle instability while fostering overall physical resilience [11].

The most striking outcome of the rehabilitation program was the remarkable reduction in pain levels, with the patient's NPRS score plummeting from 7/10 to an impressive 1/10 following the intervention. This profound decrease underscores the physical improvement and the restoration of the patient's confidence in her ankle's functionality during daily activities. Notably, the patient's performance on the CAIT score demonstrated substantial enhancement, rising from 15/30 (50%) to 28/30 (93%). This improvement reflects a considerable increase in ankle stability and the patient's psychological confidence, essential components for successful rehabilitation. Similarly, the progress observed in the FAOS from 63% to 89% further illustrates the positive trajectory in her functional status and overall quality of life. Gait analysis provided additional insights into the patient's recovery, revealing an increase in walking speed to 0.90 m/s and a cadence of 99.48 steps per minute over a 20-meter distance, achieved in just 15 seconds. These enhancements in mobility and efficiency are paramount, not only facilitating her daily routines but also fostering a more active lifestyle is an essential element in preventing future injuries. The multifaceted rehabilitation approach employed in this case is particularly noteworthy. Core and hip strengthening exercises were critical in enhancing postural control and balance, essential for maintaining ankle stability during dynamic activities [12].

Ankle-specific muscle strengthening targeted the dorsiflexors, plantar flexors, invertors, and evertors, further reinforcing joint stability and reducing future sprain risks [13]. Dynamic balance training challenged the patient's proprioception, enabling her to better navigate uneven surfaces is a vital skill for everyday safety and athletic performance [14]. Moreover, exercises focusing on the intrinsic muscles of the foot were instrumental in improving proprioception and overall stability. Research supports these interventions, indicating that core stability exercises and intrinsic foot muscle training are effective strategies for individuals with CAI [15]. According to Somayeh Alizamani et al., core stability exercises appear to enhance the measured parameters in patients with ankle instability. Consequently, this type of training is suggested as a therapeutic approach for individuals with CAI [16]. Moreover, Dong-Rour Lee suggested that exercises targeting the intrinsic foot muscles are an effective treatment for enhancing both functional performance and navigating uneven surfaces to better lance the ability of patients with chronic ankle instability, and the results were encouraging [17]. The integration of these approaches not only provides symptomatic relief but also addresses the underlying biomechanics affecting the patient's condition [18].

This case emphasizes crucial considerations for clinicians managing CAI the importance of addressing underlying foot mechanics, such as the high longitudinal arches observed in this patient, which may perpetuate instability through altered biomechanics. Although not specifically explored in this case, implementing orthotics or footwear modifications could further enhance patient outcomes.

Despite the positive results, it is essential to acknowledge the limitations inherent in this single-case report. The study focuses on short-term outcomes post-rehabilitation without addressing the long-term persistence of symptom relief. To strengthen the conclusions, it is important to acknowledge this as a limitation, specifically noting the lack of follow-up data to confirm whether symptoms reoccurred over time. This inclusion would provide a more comprehensive view of the study's findings. The findings cannot be generalized across the broader population of individuals with CAI due to the unique characteristics of the patient. In addition, the lack of a defined follow-up period poses challenges in appraising the long-term sustainability of the intervention's benefits. Therefore, future studies involving larger sample sizes and extended follow-up periods are vital to validate these findings and better understand the long-term effects of comprehensive physiotherapy on CAI.

## Conclusions

This case strongly advocates for a holistic, multidimensional approach to the management of chronic ankle instability through physiotherapy. By targeting core stability, strength, balance, and proprioception, rehabilitation can significantly improve pain, functionality, and quality of life, offering a non-surgical avenue for patients seeking relief from this often debilitating condition. The promising outcomes observed in this case point to a need for ongoing research into optimizing treatment strategies, ultimately enhancing care for individuals grappling with chronic ankle instability.
